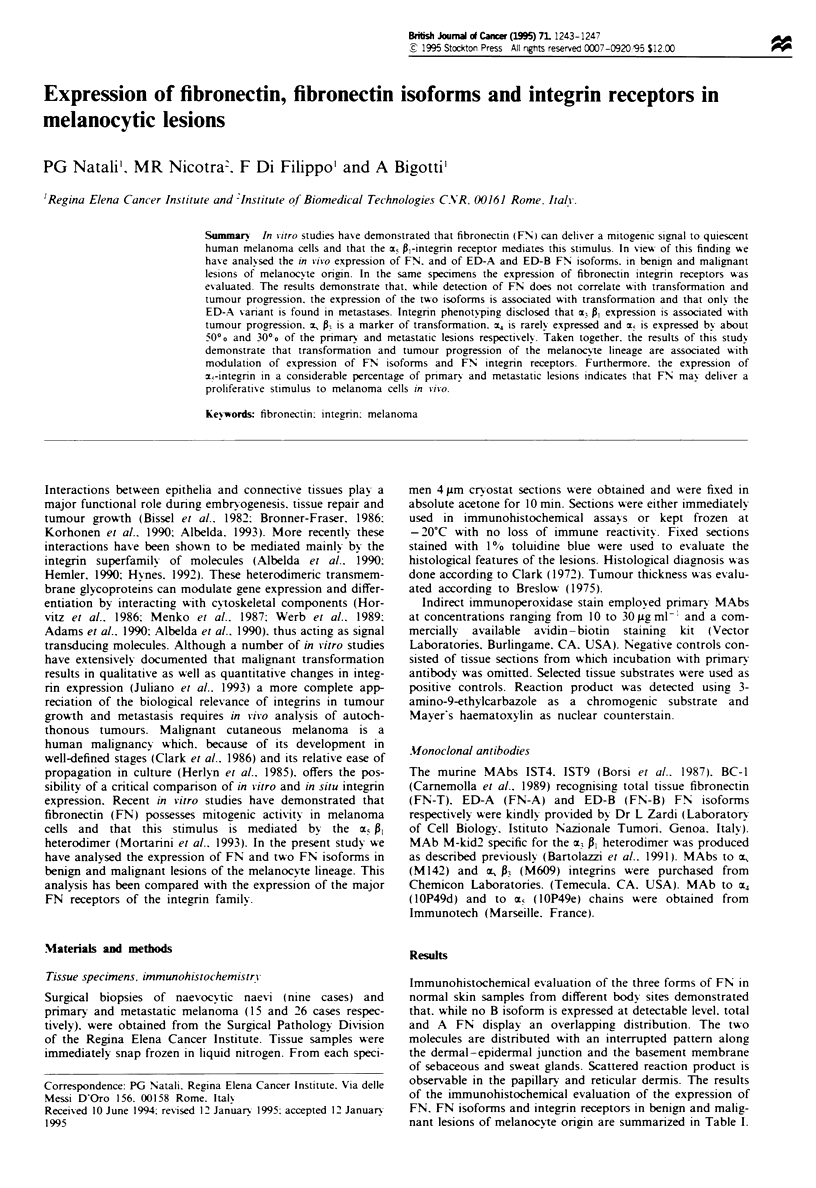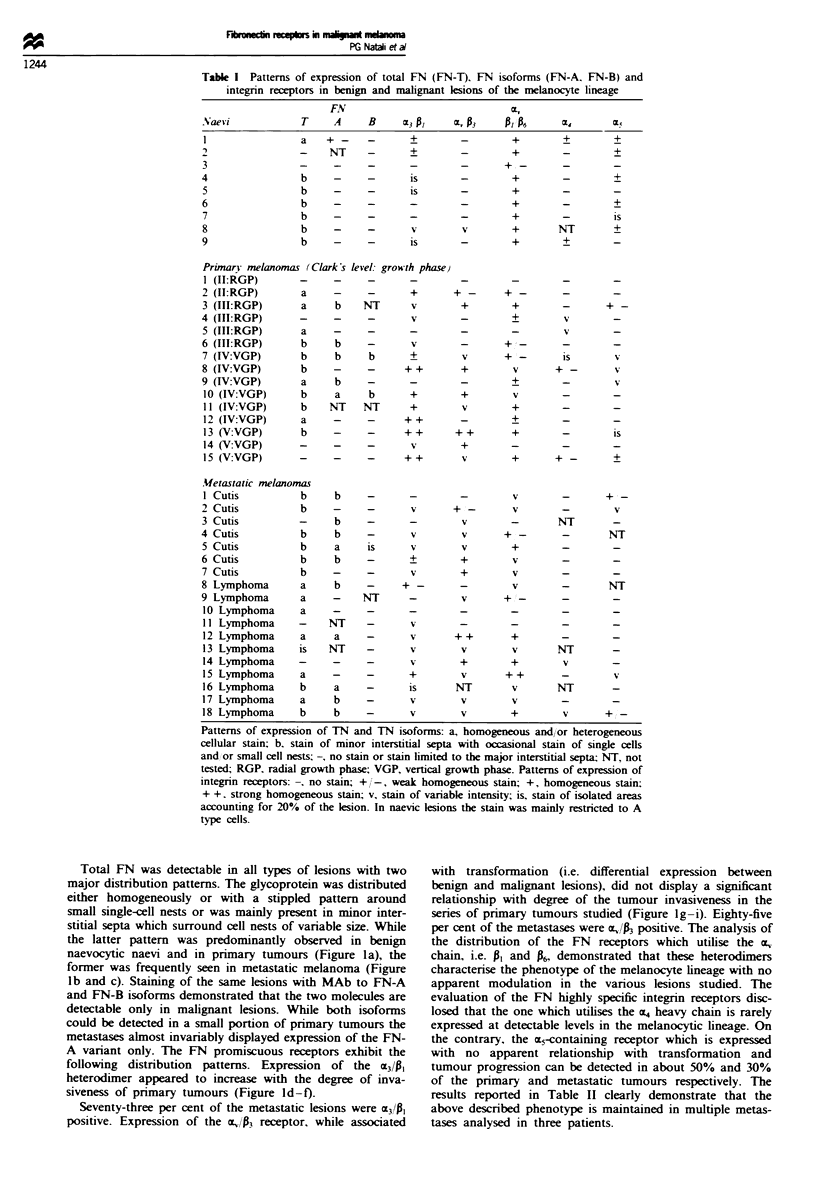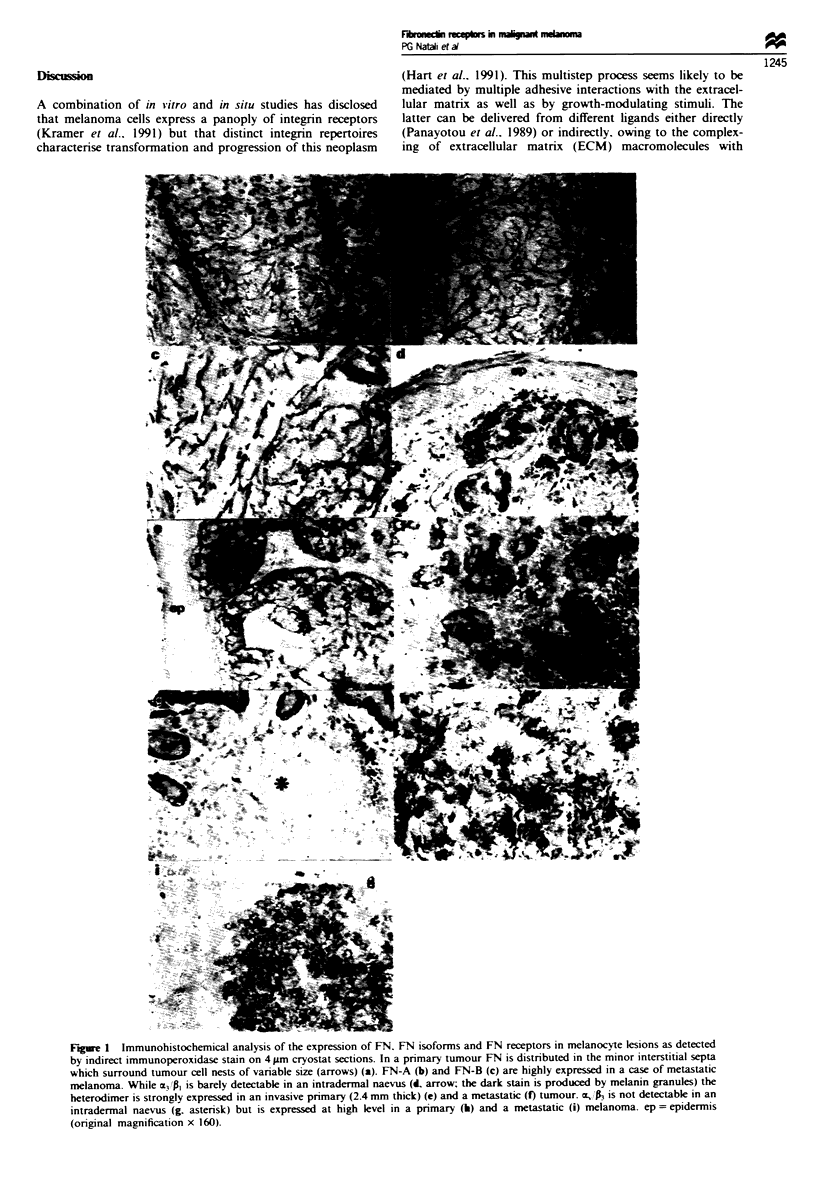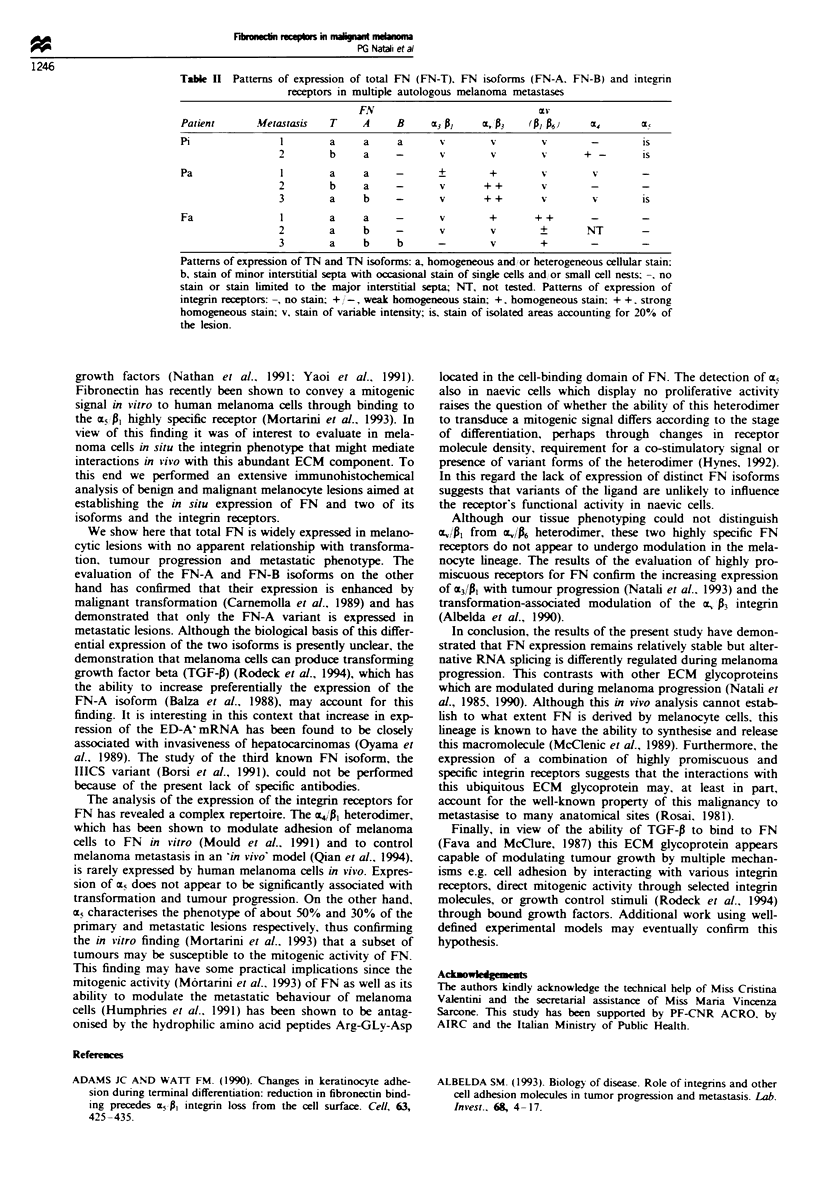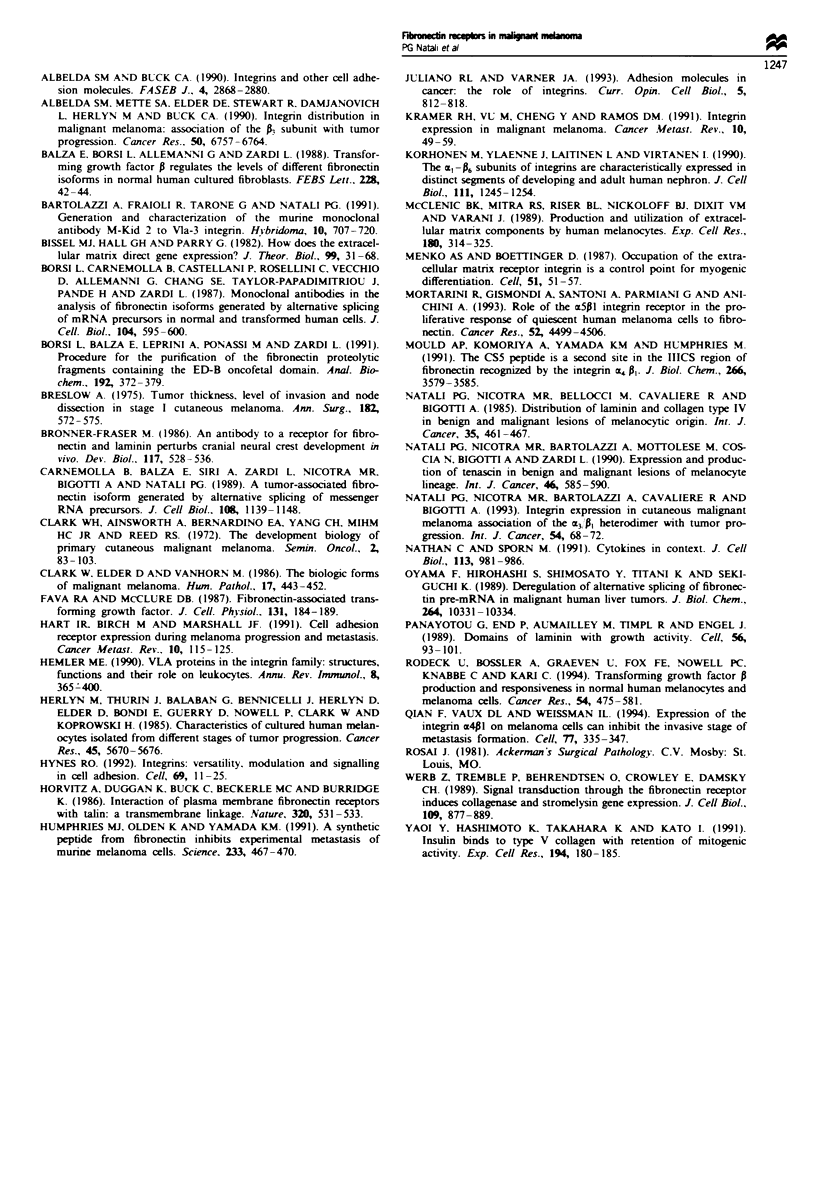# Expression of fibronectin, fibronectin isoforms and integrin receptors in melanocytic lesions.

**DOI:** 10.1038/bjc.1995.240

**Published:** 1995-06

**Authors:** P. G. Natali, M. R. Nicotra, F. Di Filippo, A. Bigotti

**Affiliations:** Regina Elena Cancer Institute, Rome, Italy.

## Abstract

**Images:**


British Journal of Cancer (1995) 71, 1243-1247

© 1995 Stockton Press All rights reserved 0007-0920/95 $12.00

### Expression of fibronectin, fibronectin isoforms and integrin receptors in melanocytic lesions

PG Natali[1], MR Nicotra[2], F Di Filippo[1] and A Bigotti[1]

[1]*Regina Elena Cancer Institute and* [2]*Institute of Biomedical Technologies CNR, 00161 Rome, Italy.*

**Summary** *In vitro* studies have demonstrated that fibronectin (FN) can deliver a mitogenic signal to quiescent human melanoma cells and that the $\alpha_5\beta_1$-integrin receptor mediates this stimulus. In view of this finding we have analysed the *in vivo* expression of FN, and of ED-A and ED-B FN isoforms, in benign and malignant lesions of melanocyte origin. In the same specimens the expression of fibronectin integrin receptors was evaluated. The results demonstrate that, while detection of FN does not correlate with transformation and tumour progression, the expression of the two isoforms is associated with transformation and that only the ED-A variant is found in metastases. Integrin phenotyping disclosed that $\alpha_3\beta_1$ expression is associated with tumour progression, $\alpha_v\beta_3$ is a marker of transformation, $\alpha_4$ is rarely expressed and $\alpha_5$ is expressed by about 50% and 30% of the primary and metastatic lesions respectively. Taken together, the results of this study demonstrate that transformation and tumour progression of the melanocyte lineage are associated with modulation of expression of FN isoforms and FN integrin receptors. Furthermore, the expression of $\alpha_5$-integrin in a considerable percentage of primary and metastatic lesions indicates that FN may deliver a proliferative stimulus to melanoma cells *in vivo*.

**Keywords:** fibronectin; integrin; melanoma

Interactions between epithelia and connective tissues play a major functional role during embryogenesis, tissue repair and tumour growth (Bissel *et al*., 1982; Bronner-Fraser, 1986; Korhonen *et al*., 1990; Albelda, 1993). More recently these interactions have been shown to be mediated mainly by the integrin superfamily of molecules (Albelda *et al*., 1990; Hemler, 1990; Hynes, 1992). These heterodimeric transmembrane glycoproteins can modulate gene expression and differentiation by interacting with cytoskeletal components (Horvitz *et al*., 1986; Menko *et al*., 1987; Werb *et al*., 1989; Adams *et al*., 1990; Albelda *et al*., 1990), thus acting as signal transducing molecules. Although a number of *in vitro* studies have extensively documented that malignant transformation results in qualitative as well as quantitative changes in integrin expression (Juliano *et al*., 1993) a more complete appreciation of the biological relevance of integrins in tumour growth and metastasis requires *in vivo* analysis of autochthonous tumours. Malignant cutaneous melanoma is a human malignancy which, because of its development in well-defined stages (Clark *et al*., 1986) and its relative ease of propagation in culture (Herlyn *et al*., 1985), offers the possibility of a critical comparison of *in vitro* and *in situ* integrin expression. Recent *in vitro* studies have demonstrated that fibronectin (FN) possesses mitogenic activity in melanoma cells and that this stimulus is mediated by the $\alpha_5\beta_1$ heterodimer (Mortarini *et al*., 1993). In the present study we have analysed the expression of FN and two FN isoforms in benign and malignant lesions of the melanocyte lineage. This analysis has been compared with the expression of the major FN receptors of the integrin family.

### Materials and methods

### *Tissue specimens, immunohistochemistry*

Surgical biopsies of naevocytic naevi (nine cases) and primary and metastatic melanoma (15 and 26 cases respectively), were obtained from the Surgical Pathology Division of the Regina Elena Cancer Institute. Tissue samples were immediately snap frozen in liquid nitrogen. From each specimen 4 μm cryostat sections were obtained and were fixed in absolute acetone for 10 min. Sections were either immediately used in immunohistochemical assays or kept frozen at −20°C with no loss of immune reactivity. Fixed sections stained with 1% toluidine blue were used to evaluate the histological features of the lesions. Histological diagnosis was done according to Clark (1972). Tumour thickness was evaluated according to Breslow (1975).

Indirect immunoperoxidase stain employed primary MAbs at concentrations ranging from 10 to 30 μg ml$^{-1}$ and a commercially available avidin–biotin staining kit (Vector Laboratories, Burlingame, CA, USA). Negative controls consisted of tissue sections from which incubation with primary antibody was omitted. Selected tissue substrates were used as positive controls. Reaction product was detected using 3-amino-9-ethylcarbazole as a chromogenic substrate and Mayer's haematoxylin as nuclear counterstain.

### *Monoclonal antibodies*

The murine MAbs IST4, IST9 (Borsi *et al*., 1987), BC-1 (Carnemolla *et al*., 1989) recognising total tissue fibronectin (FN-T), ED-A (FN-A) and ED-B (FN-B) FN isoforms respectively were kindly provided by Dr L Zardi (Laboratory of Cell Biology, Istituto Nazionale Tumori, Genoa, Italy). MAb M-kid2 specific for the $\alpha_3\beta_1$ heterodimer was produced as described previously (Bartolazzi *et al*., 1991). MAbs to $\alpha_v$ (M142) and $\alpha_v\beta_3$ (M609) integrins were purchased from Chemicon Laboratories, (Temecula, CA, USA). MAb to $\alpha_4$ (10P49d) and to $\alpha_5$ (10P49e) chains were obtained from Immunotech (Marseille, France).

### Results

Immunohistochemical evaluation of the three forms of FN in normal skin samples from different body sites demonstrated that, while no B isoform is expressed at detectable level, total and A FN display an overlapping distribution. The two molecules are distributed with an interrupted pattern along the dermal–epidermal junction and the basement membrane of sebaceous and sweat glands. Scattered reaction product is observable in the papillary and reticular dermis. The results of the immunohistochemical evaluation of the expression of FN, FN isoforms and integrin receptors in benign and malignant lesions of melanocyte origin are summarized in Table I.

Correspondence: PG Natali, Regina Elena Cancer Institute, Via delle Messi D'Oro 156, 00158 Rome, Italy
Received 10 June 1994; revised 12 January 1995; accepted 12 January 1995

**Table I** Patterns of expression of total FN (FN-T), FN isoforms (FN-A, FN-B) and integrin receptors in benign and malignant lesions of the melanocyte lineage

| | FN | | | | | $\alpha_v$ | | |
|---|---|---|---|---|---|---|---|---|
| *Naevi* | *T* | *A* | *B* | $\alpha_3\beta_1$ | $\alpha_v\beta_3$ | $\beta_1/\beta_6$ | $\alpha_4$ | $\alpha_5$ |
| 1 | a | + − | − | ± | − | + | ± | ± |
| 2 | − | NT | − | ± | − | + | − | ± |
| 3 | − | − | − | − | − | +/− | − | − |
| 4 | b | − | − | is | − | + | − | ± |
| 5 | b | − | − | is | − | + | − | − |
| 6 | b | − | − | − | − | + | − | ± |
| 7 | b | − | − | − | − | + | − | is |
| 8 | b | − | − | v | v | + | NT | ± |
| 9 | b | − | − | is | − | + | ± | − |
| *Primary melanomas (Clark's level: growth phase)* | | | | | | | | |
| 1 (II:RGP) | − | − | − | − | − | − | − | − |
| 2 (II:RGP) | a | − | − | + | +/− | +/− | − | − |
| 3 (III:RGP) | a | b | NT | v | + | + | − | + − |
| 4 (III:RGP) | − | − | − | v | − | ± | v | − |
| 5 (III:RGP) | a | − | − | − | − | − | v | − |
| 6 (III:RGP) | b | b | − | v | − | +/− | − | − |
| 7 (IV:VGP) | b | b | b | ± | v | +/− | is | v |
| 8 (IV:VGP) | b | − | − | ++ | + | v | + − | v |
| 9 (IV:VGP) | a | b | − | − | − | ± | − | v |
| 10 (IV:VGP) | b | a | b | + | + | v | − | − |
| 11 (IV:VGP) | b | NT | NT | + | v | + | − | − |
| 12 (IV:VGP) | a | − | − | ++ | − | ± | − | − |
| 13 (V:VGP) | b | − | − | ++ | ++ | + | − | is |
| 14 (V:VGP) | − | − | − | v | + | − | − | − |
| 15 (V:VGP) | − | − | − | ++ | v | + | + − | ± |
| *Metastatic melanomas* | | | | | | | | |
| 1 Cutis | b | b | − | − | − | v | − | +/− |
| 2 Cutis | b | − | − | v | +/− | v | − | v |
| 3 Cutis | − | b | − | − | v | − | NT | − |
| 4 Cutis | b | b | − | v | v | +/− | − | NT |
| 5 Cutis | b | a | is | v | v | + | − | − |
| 6 Cutis | b | b | − | ± | + | v | − | − |
| 7 Cutis | b | − | − | v | + | v | − | − |
| 8 Lymphoma | a | b | − | + − | − | v | − | NT |
| 9 Lymphoma | a | − | NT | − | v | +/− | − | − |
| 10 Lymphoma | a | − | − | − | − | − | − | − |
| 11 Lymphoma | − | NT | − | v | − | − | − | − |
| 12 Lymphoma | a | a | − | v | ++ | + | − | − |
| 13 Lymphoma | is | NT | − | v | v | v | NT | − |
| 14 Lymphoma | − | − | − | v | + | + | v | − |
| 15 Lymphoma | a | − | − | + | v | ++ | − | v |
| 16 Lymphoma | b | a | − | is | NT | v | NT | − |
| 17 Lymphoma | a | b | − | v | v | v | − | − |
| 18 Lymphoma | b | b | − | v | v | + | v | +/− |

Patterns of expression of TN and TN isoforms: a, homogeneous and/or heterogeneous cellular stain; b, stain of minor interstitial septa with occasional stain of single cells and/or small cell nests; −, no stain or stain limited to the major interstitial septa; NT, not tested; RGP, radial growth phase; VGP, vertical growth phase. Patterns of expression of integrin receptors: −, no stain; +/−, weak homogeneous stain; +, homogeneous stain; + +, strong homogeneous stain; v, stain of variable intensity; is, stain of isolated areas accounting for 20% of the lesion. In naevic lesions the stain was mainly restricted to A type cells.

Total FN was detectable in all types of lesions with two major distribution patterns. The glycoprotein was distributed either homogeneously or with a stippled pattern around small single-cell nests or was mainly present in minor interstitial septa which surround cell nests of variable size. While the latter pattern was predominantly observed in benign naevocytic naevi and in primary tumours (Figure 1a), the former was frequently seen in metastatic melanoma (Figure 1b and c). Staining of the same lesions with MAb to FN-A and FN-B isoforms demonstrated that the two molecules are detectable only in malignant lesions. While both isoforms could be detected in a small portion of primary tumours the metastases almost invariably displayed expression of the FN-A variant only. The FN promiscuous receptors exhibit the following distribution patterns. Expression of the $\alpha_3/\beta_1$ heterodimer appeared to increase with the degree of invasiveness of primary tumours (Figure 1d–f).

Seventy-three per cent of the metastatic lesions were $\alpha_3/\beta_1$ positive. Expression of the $\alpha_v/\beta_3$ receptor, while associated with transformation (i.e. differential expression between benign and malignant lesions), did not display a significant relationship with degree of the tumour invasiveness in the series of primary tumours studied (Figure 1g–i). Eighty-five per cent of the metastases were $\alpha_v/\beta_3$ positive. The analysis of the distribution of the FN receptors which utilise the $\alpha_v$ chain, i.e. $\beta_1$ and $\beta_6$, demonstrated that these heterodimers characterise the phenotype of the melanocyte lineage with no apparent modulation in the various lesions studied. The evaluation of the FN highly specific integrin receptors disclosed that the one which utilises the $\alpha_4$ heavy chain is rarely expressed at detectable levels in the melanocytic lineage. On the contrary, the $\alpha_5$-containing receptor which is expressed with no apparent relationship with transformation and tumour progression can be detected in about 50% and 30% of the primary and metastatic tumours respectively. The results reported in Table II clearly demonstrate that the above described phenotype is maintained in multiple metastases analysed in three patients.

### Discussion

A combination of *in vitro* and *in situ* studies has disclosed that melanoma cells express a panoply of integrin receptors (Kramer *et al.*, 1991) but that distinct integrin repertoires characterise transformation and progression of this neoplasm (Hart *et al.*, 1991). This multistep process seems likely to be mediated by multiple adhesive interactions with the extracellular matrix as well as by growth-modulating stimuli. The latter can be delivered from different ligands either directly (Panayotou *et al.*, 1989) or indirectly, owing to the complexing of extracellular matrix (ECM) macromolecules with

**Figure 1** Immunohistochemical analysis of the expression of FN, FN isoforms and FN receptors in melanocyte lesions as detected by indirect immunoperoxidase stain on 4 μm cryostat sections. In a primary tumour FN is distributed in the minor interstitial septa which surround tumour cell nests of variable size (arrows) (**a**). FN-A (**b**) and FN-B (**c**) are highly expressed in a case of metastatic melanoma. While $\alpha_5/\beta_1$ is barely detectable in an intradermal naevus (**d**, arrow; the dark stain is produced by melanin granules) the heterodimer is strongly expressed in an invasive primary (2.4 mm thick) (**e**) and a metastatic (**f**) tumour. $\alpha_v/\beta_3$ is not detectable in an intradermal naevus (**g**, asterisk) but is expressed at high level in a primary (**h**) and a metastatic (**i**) melanoma. ep = epidermis (original magnification × 160).

**Table II** Patterns of expression of total FN (FN-T), FN isoforms (FN-A, FN-B) and integrin receptors in multiple autologous melanoma metastases

| | | *FN* | | | | | $\alpha v$ | | |
|---|---|---|---|---|---|---|---|---|---|
| *Patient* | *Metastasis* | *T* | *A* | *B* | $\alpha_3/\beta_1$ | $\alpha_v/\beta_3$ | $(\beta_1/\beta_6)$ | $\alpha_4$ | $\alpha_5$ |
| Pi | 1 | a | a | a | v | v | v | – | is |
| | 2 | b | a | – | v | v | v | + – | is |
| Pa | 1 | a | a | – | ± | + | v | v | – |
| | 2 | b | a | – | v | + + | v | – | – |
| | 3 | a | b | – | v | + + | v | v | is |
| Fa | 1 | a | a | – | v | + | + + | – | – |
| | 2 | a | b | – | v | v | ± | NT | – |
| | 3 | a | b | b | – | v | + | – | – |

Patterns of expression of TN and TN isoforms: a, homogeneous and/or heterogeneous cellular stain; b, stain of minor interstitial septa with occasional stain of single cells and/or small cell nests; –, no stain or stain limited to the major interstitial septa; NT, not tested. Patterns of expression of integrin receptors: –, no stain; +/–, weak homogeneous stain; +, homogeneous stain; + +, strong homogeneous stain; v, stain of variable intensity; is, stain of isolated areas accounting for 20% of the lesion.

growth factors (Nathan *et al*., 1991; Yaoi *et al*., 1991). Fibronectin has recently been shown to convey a mitogenic signal *in vitro* to human melanoma cells through binding to the $\alpha_5/\beta_1$ highly specific receptor (Mortarini *et al*., 1993). In view of this finding it was of interest to evaluate in melanoma cells *in situ* the integrin phenotype that might mediate interactions *in vivo* with this abundant ECM component. To this end we performed an extensive immunohistochemical analysis of benign and malignant melanocyte lesions aimed at establishing the *in situ* expression of FN and two of its isoforms and the integrin receptors.

We show here that total FN is widely expressed in melanocytic lesions with no apparent relationship with transformation, tumour progression and metastatic phenotype. The evaluation of the FN-A and FN-B isoforms on the other hand has confirmed that their expression is enhanced by malignant transformation (Carnemolla *et al*., 1989) and has demonstrated that only the FN-A variant is expressed in metastatic lesions. Although the biological basis of this differential expression of the two isoforms is presently unclear, the demonstration that melanoma cells can produce transforming growth factor beta (TGF-$\beta$) (Rodeck *et al*., 1994), which has the ability to increase preferentially the expression of the FN-A isoform (Balza *et al*., 1988), may account for this finding. It is interesting in this context that increase in expression of the ED-A$^+$ mRNA has been found to be closely associated with invasiveness of hepatocarcinomas (Oyama *et al*., 1989). The study of the third known FN isoform, the IIICS variant (Borsi *et al*., 1991), could not be performed because of the present lack of specific antibodies.

The analysis of the expression of the integrin receptors for FN has revealed a complex repertoire. The $\alpha_4/\beta_1$ heterodimer, which has been shown to modulate adhesion of melanoma cells to FN *in vitro* (Mould *et al*., 1991) and to control melanoma metastasis in an '*in vivo*' model (Qian *et al*., 1994), is rarely expressed by human melanoma cells *in vivo*. Expression of $\alpha_5$ does not appear to be significantly associated with transformation and tumour progression. On the other hand, $\alpha_5$ characterises the phenotype of about 50% and 30% of the primary and metastatic lesions respectively, thus confirming the *in vitro* finding (Mortarini *et al*., 1993) that a subset of tumours may be susceptible to the mitogenic activity of FN. This finding may have some practical implications since the mitogenic activity (Mortarini *et al*., 1993) of FN as well as its ability to modulate the metastatic behaviour of melanoma cells (Humphries *et al*., 1991) has been shown to be antagonised by the hydrophilic amino acid peptides Arg-GLy-Asp located in the cell-binding domain of FN. The detection of $\alpha_5$ also in naevic cells which display no proliferative activity raises the question of whether the ability of this heterodimer to transduce a mitogenic signal differs according to the stage of differentiation, perhaps through changes in receptor molecule density, requirement for a co-stimulatory signal or presence of variant forms of the heterodimer (Hynes, 1992). In this regard the lack of expression of distinct FN isoforms suggests that variants of the ligand are unlikely to influence the receptor's functional activity in naevic cells.

Although our tissue phenotyping could not distinguish $\alpha_v/\beta_1$ from $\alpha_v/\beta_6$ heterodimer, these two highly specific FN receptors do not appear to undergo modulation in the melanocyte lineage. The results of the evaluation of highly promiscuous receptors for FN confirm the increasing expression of $\alpha_3/\beta_1$ with tumour progression (Natali *et al*., 1993) and the transformation-associated modulation of the $\alpha_v/\beta_3$ integrin (Albelda *et al*., 1990).

In conclusion, the results of the present study have demonstrated that FN expression remains relatively stable but alternative RNA splicing is differently regulated during melanoma progression. This contrasts with other ECM glycoproteins which are modulated during melanoma progression (Natali *et al*., 1985, 1990). Although this *in vivo* analysis cannot establish to what extent FN is derived by melanocyte cells, this lineage is known to have the ability to synthesise and release this macromolecule (McClenic *et al*., 1989). Furthermore, the expression of a combination of highly promiscuous and specific integrin receptors suggests that the interactions with this ubiquitous ECM glycoprotein may, at least in part, account for the well-known property of this malignancy to metastasise to many anatomical sites (Rosai, 1981).

Finally, in view of the ability of TGF-$\beta$ to bind to FN (Fava and McClure, 1987) this ECM glycoprotein appears capable of modulating tumour growth by multiple mechanisms e.g. cell adhesion by interacting with various integrin receptors, direct mitogenic activity through selected integrin molecules, or growth control stimuli (Rodeck *et al*., 1994) through bound growth factors. Additional work using well-defined experimental models may eventually confirm this hypothesis.

**Acknowledgements**
The authors kindly acknowledge the technical help of Miss Cristina Valentini and the secretarial assistance of Miss Maria Vincenza Sarcone. This study has been supported by PF-CNR ACRO, by AIRC and the Italian Ministry of Public Health.

**References**

ADAMS JC AND WATT FM. (1990). Changes in keratinocyte adhesion during terminal differentiation: reduction in fibronectin binding precedes $\alpha_5/\beta_1$ integrin loss from the cell surface. *Cell*, **63**, 425-435.

ALBELDA SM. (1993). Biology of disease. Role of integrins and other cell adhesion molecules in tumor progression and metastasis. *Lab. Invest.*, **68**, 4-17.

ALBELDA SM AND BUCK CA. (1990). Integrins and other cell adhesion molecules. *FASEB J.*, **4**, 2868-2880.

ALBELDA SM, METTE SA, ELDER DE, STEWART R, DAMJANOVICH L, HERLYN M AND BUCK CA. (1990). Integrin distribution in malignant melanoma: association of the $\beta_3$ subunit with tumor progression. *Cancer Res.*, **50**, 6757-6764.

BALZA E, BORSI L, ALLEMANNI G AND ZARDI L. (1988). Transforming growth factor $\beta$ regulates the levels of different fibronectin isoforms in normal human cultured fibroblasts. *FEBS Lett.*, **228**, 42-44.

BARTOLAZZI A, FRAIOLI R, TARONE G AND NATALI PG. (1991). Generation and characterization of the murine monoclonal antibody M-Kid 2 to Vla-3 integrin. *Hybridoma*, **10**, 707-720.

BISSEL MJ, HALL GH AND PARRY G. (1982). How does the extracellular matrix direct gene expression? *J. Theor. Biol.*, **99**, 31-68.

BORSI L, CARNEMOLLA B, CASTELLANI P, ROSELLINI C, VECCHIO D, ALLEMANNI G, CHANG SE, TAYLOR-PAPADIMITRIOU J, PANDE H AND ZARDI L. (1987). Monoclonal antibodies in the analysis of fibronectin isoforms generated by alternative splicing of mRNA precursors in normal and transformed human cells. *J. Cell. Biol.*, **104**, 595-600.

BORSI L, BALZA E, LEPRINI A, PONASSI M AND ZARDI L. (1991). Procedure for the purification of the fibronectin proteolytic fragments containing the ED-B oncofetal domain. *Anal. Biochem.*, **192**, 372-379.

BRESLOW A. (1975). Tumor thickness, level of invasion and node dissection in stage I cutaneous melanoma. *Ann. Surg.*, **182**, 572-575.

BRONNER-FRASER M. (1986). An antibody to a receptor for fibronectin and laminin perturbs cranial neural crest development *in vivo*. *Dev. Biol.*, **117**, 528-536.

CARNEMOLLA B, BALZA E, SIRI A, ZARDI L, NICOTRA MR, BIGOTTI A AND NATALI PG. (1989). A tumor-associated fibronectin isoform generated by alternative splicing of messenger RNA precursors. *J. Cell Biol.*, **108**, 1139-1148.

CLARK WH, AINSWORTH A, BERNARDINO EA, YANG CH, MIHM HC JR AND REED RS. (1972). The development biology of primary cutaneous malignant melanoma. *Semin. Oncol.*, **2**, 83-103.

CLARK W, ELDER D AND VANHORN M. (1986). The biologic forms of malignant melanoma. *Hum. Pathol.*, **17**, 443-452.

FAVA RA AND McCLURE DB. (1987). Fibronectin-associated transforming growth factor. *J. Cell. Physiol.*, **131**, 184-189.

HART IR, BIRCH M AND MARSHALL JF. (1991). Cell adhesion receptor expression during melanoma progression and metastasis. *Cancer Metast. Rev.*, **10**, 115-125.

HEMLER ME. (1990). VLA proteins in the integrin family: structures, functions and their role on leukocytes. *Annu. Rev. Immunol.*, **8**, 365-400.

HERLYN M, THURIN J, BALABAN G, BENNICELLI J, HERLYN D, ELDER D, BONDI E, GUERRY D, NOWELL P, CLARK W AND KOPROWSKI H. (1985). Characteristics of cultured human melanocytes isolated from different stages of tumor progression. *Cancer Res.*, **45**, 5670-5676.

HYNES RO. (1992). Integrins: versatility, modulation and signalling in cell adhesion. *Cell*, **69**, 11-25.

HORVITZ A, DUGGAN K, BUCK C, BECKERLE MC AND BURRIDGE K. (1986). Interaction of plasma membrane fibronectin receptors with talin: a transmembrane linkage. *Nature*, **320**, 531-533.

HUMPHRIES MJ, OLDEN K AND YAMADA KM. (1991). A synthetic peptide from fibronectin inhibits experimental metastasis of murine melanoma cells. *Science*, **233**, 467-470.

JULIANO RL AND VARNER JA. (1993). Adhesion molecules in cancer: the role of integrins. *Curr. Opin. Cell Biol.*, **5**, 812-818.

KRAMER RH, VU M, CHENG Y AND RAMOS DM. (1991). Integrin expression in malignant melanoma. *Cancer Metast. Rev.*, **10**, 49-59.

KORHONEN M, YLAENNE J, LAITINEN L AND VIRTANEN I. (1990). The $\alpha_1-\beta_6$ subunits of integrins are characteristically expressed in distinct segments of developing and adult human nephron. *J. Cell Biol.*, **111**, 1245-1254.

McCLENIC BK, MITRA RS, RISER BL, NICKOLOFF BJ, DIXIT VM AND VARANI J. (1989). Production and utilization of extracellular matrix components by human melanocytes. *Exp. Cell Res.*, **180**, 314-325.

MENKO AS AND BOETTINGER D. (1987). Occupation of the extracellular matrix receptor integrin is a control point for myogenic differentiation. *Cell*, **51**, 51-57.

MORTARINI R, GISMONDI A, SANTONI A, PARMIANI G AND ANICHINI A. (1993). Role of the $\alpha5\beta1$ integrin receptor in the proliferative response of quiescent human melanoma cells to fibronectin. *Cancer Res.*, **52**, 4499-4506.

MOULD AP, KOMORIYA A, YAMADA KM AND HUMPHRIES M. (1991). The CS5 peptide is a second site in the IIICS region of fibronectin recognized by the integrin $\alpha_4\beta_1$. *J. Biol. Chem.*, **266**, 3579-3585.

NATALI PG, NICOTRA MR, BELLOCCI M, CAVALIERE R AND BIGOTTI A. (1985). Distribution of laminin and collagen type IV in benign and malignant lesions of melanocytic origin. *Int. J. Cancer*, **35**, 461-467.

NATALI PG, NICOTRA MR, BARTOLAZZI A, MOTTOLESE M, COSCIA N, BIGOTTI A AND ZARDI L. (1990). Expression and production of tenascin in benign and malignant lesions of melanocyte lineage. *Int. J. Cancer*, **46**, 585-590.

NATALI PG, NICOTRA MR, BARTOLAZZI A, CAVALIERE R AND BIGOTTI A. (1993). Integrin expression in cutaneous malignant melanoma association of the $\alpha_3/\beta_1$ heterodimer with tumor progression. *Int. J. Cancer*, **54**, 68-72.

NATHAN C AND SPORN M. (1991). Cytokines in context. *J. Cell Biol.*, **113**, 981-986.

OYAMA F, HIROHASHI S, SHIMOSATO Y, TITANI K AND SEKIGUCHI K. (1989). Deregulation of alternative splicing of fibronectin pre-mRNA in malignant human liver tumors. *J. Biol. Chem.*, **264**, 10331-10334.

PANAYOTOU G, END P, AUMAILLEY M, TIMPL R AND ENGEL J. (1989). Domains of laminin with growth activity. *Cell*, **56**, 93-101.

RODECK U, BOSSLER A, GRAEVEN U, FOX FE, NOWELL PC, KNABBE C AND KARI C. (1994). Transforming growth factor $\beta$ production and responsiveness in normal human melanocytes and melanoma cells. *Cancer Res.*, **54**, 475-581.

QIAN F, VAUX DL AND WEISSMAN IL. (1994). Expression of the integrin $\alpha4\beta1$ on melanoma cells can inhibit the invasive stage of metastasis formation. *Cell*, **77**, 335-347.

ROSAI J. (1981). *Ackerman's Surgical Pathology*. C.V. Mosby: St. Louis, MO.

WERB Z, TREMBLE P, BEHRENDTSEN O, CROWLEY E, DAMSKY CH. (1989). Signal transduction through the fibronectin receptor induces collagenase and stromelysin gene expression. *J. Cell Biol.*, **109**, 877-889.

YAOI Y, HASHIMOTO K, TAKAHARA K AND KATO I. (1991). Insulin binds to type V collagen with retention of mitogenic activity. *Exp. Cell Res.*, **194**, 180-185.